# Urinary KIM-1: a novel biomarker for evaluation of occupational exposure to lead

**DOI:** 10.1038/srep38930

**Published:** 2016-12-14

**Authors:** Rong Zhou, Yahong Xu, Jie Shen, Lin Han, Xi Chen, Xuefang Feng, Xingya Kuang

**Affiliations:** 1 Department of Nephrology, Yangpu Hospital, Tongji University School of Medicine, Shanghai, China; 2 Department of Occupational medicine, Yangpu Hospital, Tongji University School of Medicine, Shanghai, China

## Abstract

Chronic occult lead poisoning often develops ensuing occupational lead exposure. Early diagnosis of lead poisoning is critical for timely discontinuation of lead exposure and for prognosis. This study explored the value of urinary kidney injury molecule-1 (KIM-1) in diagnosing renal injury induced by lead at an early stage. We retrospectively analyzed 92 workers exposed to occupational lead and demonstrated a better correlation ship between blood lead levels and urine excretion of KIM-1 than other traditional renal injury biomarkers following creatinine adjustment. Receiver operating characteristic curve analysis of the ability of diverse biomarkers for predicting kidney injury in lead-exposed workers demonstrated that the order of predicting accuracy of the studied biomarkers is as follows: urinary KIM-1-to-creatinine ratio > urinary N-acetyl-*β*-(D)-glucosaminidase-to-creatinine ratio > urinary *β*2-microglobulin-to-creatinine ratio > urinary *α*1-microglobulin-to-creatinine ratio, with the Youden index being 16.59 ng/g, 14.01 U/g, 0.15 mg/g, and 4.63 mg/g, respectively. Collectively, our findings suggest that short-period occupational lead exposure may cause injury of renal tubules. Urinary excretion of KIM-1 correlates with blood lead levels better than other traditional renal injury biomarkers, including N-acetyl-*β*-(D)-glucosaminidase, *α*1-microglobulin, and *β*2-microglobulin. Longitudinal surveillance of urinary KIM-1 may aid for early diagnosis of renal tubular injury in workers with occupational lead exposure.

Lead is a naturally occurring toxic metal that widely exists in nature in trace levels but in high levels in certain occupational settings as an important source of lead poisoning. As a cumulative toxicant, lead affects multiple organ systems of the mammalian body^1^, including the kidney, brain, liver, heart and others. Lead poisoning is a severe disease that afflicts hundreds of million people worldwide and imposes a heavy burden on the treasury and health-care systems with no definitive treatment available yet. It was estimated that lead exposure accounted for 853,000 deaths in 2013 due to long-term effects on health, with the highest burden in low and middle income countries^2^. More specifically, lead exposure accounted for 9.3% of the global burden of idiopathic intellectual disability, 4% of the global burden of ischemic heart disease and 6.6% of the global burden of stroke^3^.

Lead in the body is distributed to multiple solid organs, including the brain, liver, kidney and bones. Kidney is one of the main target organs of lead toxicity^4^,^5^,^6^,^7^. Early exposure to lead can cause renal proximal tubular dysfunction, chronic interstitial nephritis and eventually irreversible progressive chronic kidney disease that culminates in end stage renal failure^8^,^9^,^10^,^11^,^12^,^13^. Therefore, early diagnosis of lead-induced renal damage is critically important for the prognosis of patients^14^,^15^. Previous studies on evaluation of lead-induced renal injury mainly focused on traditional parameters of kidney function, such as blood urea nitrogen, serum creatinine, creatinine clearance rate and low molecular weight proteinuria. However, the delayed nature of these functional indicators in revealing the kidney parenchymal impairments incurs low accuracy and poor correlation with lead induced renal damage^16^. The recently discovered kidney injury molecule-1 (KIM-1) has been shown to be an early biomarker of renal tubular injury caused by a variety of insults. So far, the application of urinary KIM-1 in evaluating lead-induced renal injury has not been explored. The current study examined the role of urinary KIM-1 in predicting lead-induced renal injury in a cohort of workers with occupation lead exposure.

## Results

### Demographic characteristics of the study subjects

**Figure 1:**
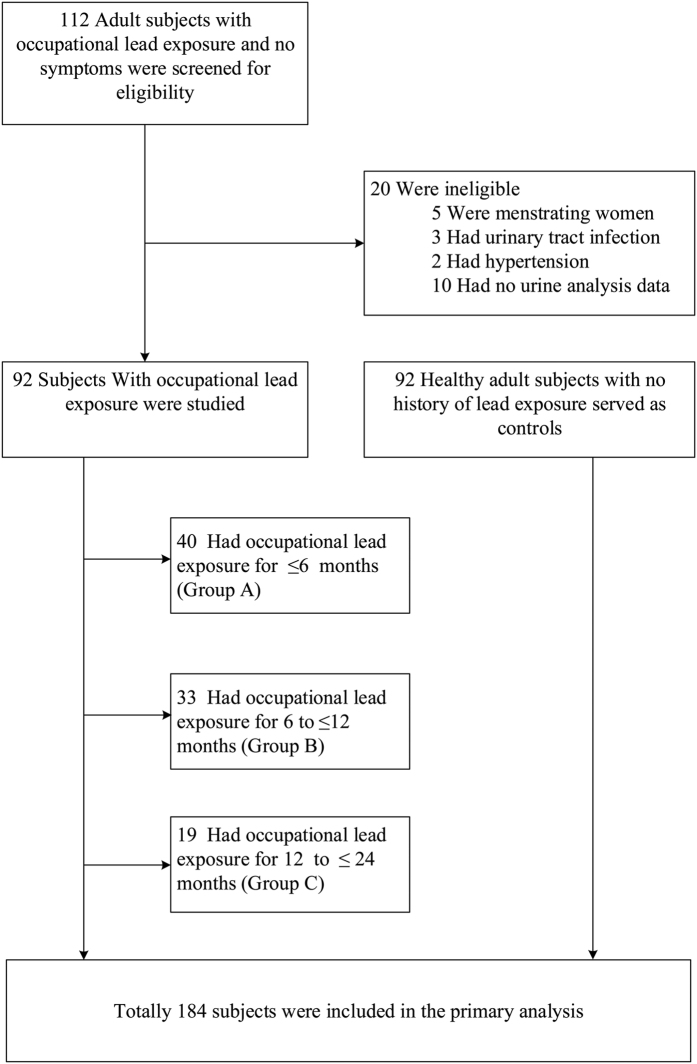
A flow diagram of the research design to evaluate urinary biomarkers for predicting kidney injury in study subjects with occupational lead exposure.

Totally 112 adult subjects that worked at a light-bulb factory and had blood lead tests were evaluated for inclusion in this study. Among them, 20 (17.9%) subjects were excluded, of which 5 were menstruating women, 3 had urinary tract infection, 2 had history of hypertension, and 10 had no urinalysis data. In addition, a separate group of 92 age- and sex-matched healthy adults were enrolled as controls (Fig. 1). Thus, the entire cohort comprised 184 subjects including 94 males (51.09%), with the ages ranging from 18 to 34 years old. The average age of the cohort was 21.02 ± 2.73 years old. The lead-exposed study cohort includes 50 males (54.3%) with the ages ranging from 18 to 34 years old and the average age being 21.20 ± 2.86 years old (Table 1). According to the length of lead exposure time, the 92 workers were categorized into 3 groups: Group A (1–6 months), group B (7~12 months), and Group C (13–24 months) (Table 1).

### Biochemical parameters and biomarkers of kidney injury

The levels of blood lead and serum cystatin C in the lead exposure group were significantly higher than those in the control group (*p* < 0.05). But the levels of blood urea nitrogen in the control group was paradoxically higher than that in lead-exposed group (*p* < 0.05) (Table 1). After correction with urinary creatinine levels, urinary excretion of kidney injury biomarkers, such as KIM-1, N-acetyl-*β*-(D)-glucosaminidase (NAG), *α*1-microglobulin (*α*1-MG), and *β*2-microglobulin (*β*2-MG), in the lead exposure group were significantly higher than those in the control group (*p* < 0.05) (Table 1).

### Urinary biomarkers of kidney injury in study subjects with varying lead exposure time

Compared with the controls, urinary KIM-1 to creatinine ratios (KIM-1/Cr), NAG to creatinine ratios (NAG/Cr), and *β*2-MG to creatinine ratios (*β*2-MG/Cr) in all subgroups of lead-exposed subjects increased significantly (*p* < 0.05). In contrast, urinary *α*1-MG to creatinine ratios (*α*1-MG/Cr) were only higher in those workers in Groups B and C that had longer period of lead exposure (*p* < 0.05). There was no statistical difference in blood lead levels among all subgroups (*p* > 0.05) (Table 2).

### Correlation between blood lead levels and urinary excretion of biomarkers of kidney injury

Spearman linear correlation analysis showed that blood lead level is positively correlated with urinary excretion of biomarkers of kidney injury, including urinary KIM-1 to creatinine (KIM-1/Cr), NAG to creatinine (NAG/Cr), *α*1-MG to creatinine (*α*1-MG/Cr), *β*2-MG to creatinine ratios (*β*2-MG/Cr), as well as serum levels of creatinine and cystatin C in a linear manner (*p* < 0.05) (Table 3).

### The predicting power of urinary biomarkers for diagnosing kidney injury following lead exposure

**Figure 2:**
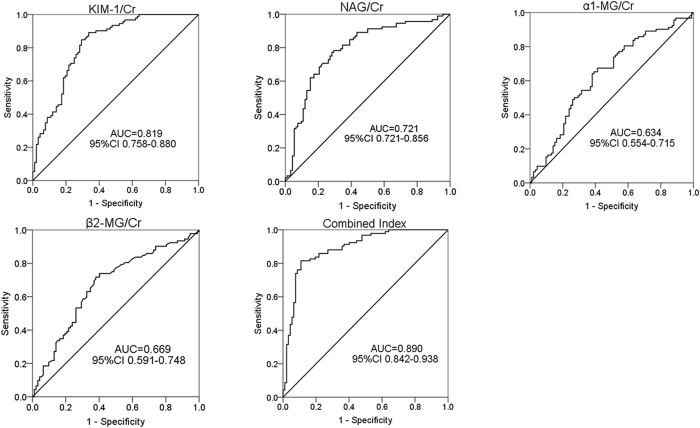
Receiver operating characteristic analysis of the predicting power of urinary biomarkers for diagnosing kidney injury in lead-exposed subjects. Abbreviations: *α*1-MG, *α*1-microglobulin; *β*2-MG, *β*2-microglobulin; Cr, creatinine; KIM-1, kidney injury molecular 1; NAG, N-acetyl-*β*-(D)-glucosaminidase. Note: Combined Index includes urinary KIM-1/Cr, urinary NAG/Cr, urinary α1-MG/Cr and urinary β2-MG/Cr.

Receiver operating characteristic (ROC) curve analysis was performed to determine the ability of diverse urinary proteins to serve as biomarkers for predicting kidney injury following lead exposure. The area under the ROC curve (AUC) was measured to evaluate the sensitivity and specificity of the urinary biomarkers. To this end, the orders of the predicting power of various urinary biomarkers for diagnosing renal tubular injury in lead-exposed subjects is as follows: KIM-1-to-creatinine ratios (KIM-1/Cr) > NAG-to-creatinine ratios (NAG/Cr) > *β*2-MG-to-creatinine ratios (*β*2-MG/Cr) > *α*1-MG-to-creatinine ratios (*α*1-MG/Cr) (Fig. 2). The Youden indexes of above urinary biomarkers were 16.59 ng/g, 14.01 U/g, 0.15 mg/g, and 4.63 mg/g, respectively (Table 4). All of the above urinary indexes were fitted by Binary Logistic Regression and in aggregate considered as the combined index. The prediction power of the combined index was the highest according to ROC analysis, followed by corroboration by Kolmogorov-Smirnov Z test (*p* < 0.05) (Table 5).

## Discussion

Heavy metal poisoning has been a focus of public health research^4^,^12^,^17^,^18^. As one of the most easily accumulated poisonous substances in human body, lead exposure is closely related to chronic renal injury^10^,^11^,^19^,^20^. Early diagnosis of lead-induced kidney damage still lacks appropriate methods with great accuracy and precision, but is critical for long term prognosis in workers with occupational lead exposure^14^,^21^,^22^. The current study examined healthy workers with occupational lead exposure and compared the traditional urinary biomarkers with KIM-1 to evaluate their ability in predicting the lead-induced renal injury.

KIM-1 is one of type I transmembrane protein that belongs to the immunoglobulin superfamily. It is barely expressed in normal kidney. The increased expression of KIM-1 in proximal tubular epithelial cells is always suggestive of acute renal injury^23^,^24^,^25^,^26^. Previous studies have also confirmed KIM-1 overexpression in a variety of acute and chronic renal injury in both experimental and human kidney diseases^27^,^28^,^29^,^30^. Since the expression levels of KIM-1 in urinary exfoliated cells are closely associated with its expression in kidney tissues, urinary KIM-1 has been employed to reflect proximal renal tubule injury. Currently, KIM-1 has become one of the key biomarkers for diagnosis, disease activity monitoring and prognosis of acute kidney injury^29^,^30^,^31^,^32^. There is evidence suggesting that KIM-1 can be used for early screening and diagnosis of renal injury induced by cadmium poisoning^33^,^34^,^35^. However, no clinical data is available assessing the correlation of urinary KIM-1 with lead-induced renal damage.

Urinary kidney biomarkers such as NAG, *β*2-MG, *α*1-MG are commonly regarded as indexes of renal tubular injury, and are always used as diagnostic parameters in renal injury elicited by heavy metals^14^. The average blood lead level in the lead-exposed subjects in the current study was 121 *μ*g/L, which is far lower than the diagnostic criteria of lead poisoning, suggestive of an early and subclinical stage of lead poisoning. Despite this, urinary excretion of *α*1-MG, *β*2-MG and NAG was much higher than that in the control group, entailing that even if the lead-exposed subjects have not shown obvious symptoms of renal impairments, like increased serum creatinine, the damage of renal tubular has occurred. Urinary levels of KIM-1 were likewise significantly higher in the lead-exposed group than those in the control group. More importantly, urinary KIM-1 demonstrated a much greater correlationship with blood lead levels than other traditional urinary biomarkers of kidney injury. The power of urinary KIM-1 in predicting renal tubular injury was also much better than *α*1-MG, *β*2-MG and NAG in the subjects exposed to occupational lead. Furthermore, combination of all above urinary indexes provided the greatest ability in evaluating the renal tubular injury as compared with KIM-1/Cr alone. Collectively, urinary KIM-1 is likely the most ideal urinary biomarker of renal tubular injury in subjects exposed to occupational lead.

In the in-depth study of workers with varying lead exposure time, we found that there was no significant difference in urinary KIM-1 levels among different subgroups. Similar results were also found in the traditional urinary biomarkers. But our data indicated that even in workers with very short period of lead exposure that was less than 6 months, urinary excretion of most biomarkers had elevated, suggestive of renal parenchymal injury at a very early stage of lead exposure. With the prolonged exposure to occupational lead, the urinary kidney biomarkers did not increase markedly. Therefore, early detection of urinary kidney biomarkers may aid for early diagnosis of renal tubular injury in lead-exposed people and for improving the outcome of kidney injury.

One of the limitations of this study is the lack of long-term follow-up of this cohort of lead-exposed subjects. In particular, it remains unknown whether urinary KIM-1 levels in lead-exposed workers continue to be higher than normal or recede after they change their jobs. Future studies are also merited to determine the incidence of acute kidney injury in the population exposed to occupational lead, to decipher the longitudinal changes of urinary biomarkers of tubular injury, and to validate the importance of the early detection of urinary kidney injury biomarkers in the population exposed to occupational lead. Moreover, it remains to be clarified why blood urea nitrogen levels at the early stage of lead exposure were lower than normal levels, as shown in our study. Probably, impaired reabsorption of urea nitrogen by the damaged renal proximal tubules may contribute, in addition to other confounding factors, such as possible less dietary protein and nitrogen intake in the socially-economically disadvantaged light-bulb labors. Another interesting finding of this study is that serum Cystatin C levels were elevated at the early stage occupational lead exposure, when serum creatinine levels were still normal. Serum Cystatin C has been suggested as an important and sensitive glomerular injury index in subjects exposed to occupational lead. A multicenter, prospective and larger epidemiology study is absolutely needed to verify the above findings.

In summary, lead exposure for a short period of time resulted in renal tubular injury. Urinary KIM-1 demonstrated a better correlationship with blood lead levels than other traditional urinary biomarkers of kidney injury, including NAG, *α*1-MG, *β*2-MG. Timely detection of urinary KIM-1 may aid for early diagnosis of renal tubular injury in workers with occupational lead exposure.

## Patients and Methods

### Study Subjects

This study conformed to the ethical guidelines of the 1975 Declaration of Helsinki and was approved by the Institutional Review Board and ethnic committee of the Yangpu Hospital of Tongji University. Written informed consents were obtained from the study subject. We retrospectively analyzed the medical records of 92 staff workers at a light bulb manufacturing plant and enrolled 92 healthy people without occupational lead exposure as the control group from July 2010 to October 2013. To be eligible for this study, study subjects must meet the following criteria: (1) adult subjects older than 18 years old but less than 65 years old; (2) no history or symptoms of acute and chronic renal injury. The exclusion criteria: (1) symptoms of acute infection and inflammation; (2) history of cancer; (3) family history of mental abnormality. (4) abnormal liver function; (5) history of hypertension; (6) history of diabetic mellitus; (7) women during menstrual periods.

### Research Protocols

The 92 workers were divided into 3 groups according to the length of lead exposure: Group A (1–6 months), group B (7~12 months), and Group C (13–24 months). Additional 92 healthy people without history of occupational lead exposure served as controls. We retrospectively analyzed the correlation among parameters such as urinary KIM-1, blood lead, blood creatinine, blood uric acid, blood cystatin C (Cys C), urinary albumin (ALB), urine *α*1-microglobulin (*α*1-MG), urine *β*2-microglobulin (*β*2-MG), urinary N- acetyl-*β*-D- glucosamine glucosidase (NAG), etc.

### Laboratory Tests

The subjects were fasted for over 12 h before collection of 5 ml venous blood, with serum separated within 3 h. Early morning urine samples were collected. Blood lead was measured by graphite probe furnace atomic absorption spectrometry. Serum creatinine was measured by enzymatic method (Beckman Coulter, US). Urinary albumin (ALB), urinary α1-microglobulin (α1-MG), and urinary β2-microglobulin (β2-MG)) were measured by immune-scatter turbidimetry (Siemens, Germany). Serum cystatin C (Cys C) was measured by colloidal gold particles immunoturbidimetry (Zhongyuan biotechnology, China). Urinary N-acetyl-β-(D)-Glucosaminidase (NAG) were measured by kinetic method (Kuake, China). Urinary KIM-1 was detected by Elisa kit (JRDUN Biotechnology, Shanghai, China). The estimated glomerular filtration rate (eGFR) was calculated CKD-EPI formula. The CKD-EPI equation, expressed as a single equation, is GFR = 141 × min (Scr/*k*,1)^α^ × max (Scr/*k*,1)^−1.209^ × 0.993^Age^ × 1.018(if female) × 1.159(if black), where *k* is 0.7 for females and 0.9 for males, *α* is −0.329 for females and −0.411 for males, min indicates the minimum of Scr/*k* or 1 and max indicates the maximum of Scr/*k* or 1^36^.

### Statistical Analysis

All data were analyzed by SPSS for windows version 15.0 (IBM Corporation, Armonk, New York, USA). Normally distributed data were presented as mean ± SD. Non-normally distributed data were presented using median (25th, 75th percentile). The continuous variables were analyzed by t-test or one AVONA. The non-normally distributed data were analyzed using the Wilcoxon rank sum test and Kolmogorov-Smirnov Z test. Spearman correlation analysis was utilized to analyze the correlation between blood lead and other parameters. *p* < 0.05 was considered statistical significance. The difference in urinary renal injury related markers between the lead-exposed groups and control group was analyzed using Krusskal-Wallis Test or LSD Test. Operating characteristic (ROC) curves and calculated the area under the ROC curve (AUC) were used to analyze the power of urinary parameters in the evaluation of renal tubular injury.

## Additional Information

**How to cite this article**: Zhou, R. *et al*. Urinary KIM-1: a novel biomarker for evaluation of occupational exposure to lead. *Sci. Rep.* **6**, 38930; doi: 10.1038/srep38930 (2016).

**Publisher's note:** Springer Nature remains neutral with regard to jurisdictional claims in published maps and institutional affiliations.

## Figures and Tables

**Table 1:** Comparison of demographic characteristics of the study subjects.

	Control group (n = 92)	Lead-exposed group (n = 92)	*p* value
Male [Case (%)]	44 (47.8%)	50 (54.3%)	0.461
Age (year)	20.85 ± 2.61	21.2 ± 2.86	0.390
Blood lead (*μ*g/L)	33 [29, 38.75]	121 [92.5, 159]	0.001
Serum CysC (mg/L)	0.6 [0.55, 0.65]	1.03 [0.92, 1.15]	0.001
eGFR (ml/min × 1.73 m^2^)	143.53 ± 12.11	141.74 ± 10.73	0.289
Serum creatinine (*μ*mol/L)	54.33 ± 11.97	56.66 ± 7.49	0.116
Serum urea nitrogen (mmol/L)	4.38 ± 1.25	4.00 ± 1.05	0.027
Serum uric acid (*μ*mol/L)	330.87 ± 80.04	316.85 ± 65.523	0.195
Urinary ALB/Cr (mg/g)	8.5 [5.9, 14.2]	10.5 [6.9, 18.1]	0.124
Urinary KIM-1/Cr (ng/g)	13.85 [9.20, 20.84]	30.02 [19.86, 50.47]	0.001
Urinary NAG/Cr (U/g)	13.10 ± 5.98	19.66 ± 8.03	0.001
Urinary *α*1-MG/Cr (mg/g)	4.35 [3.07, 7.09]	6.32 [3.98, 9.02]	0.004
Urinary *β*2-MG/Cr (mg/g)	0.13 [0.10, 0.21]	0.19 [0.13, 0.32]	0.001

Note: Normally distributed data were presented as mean ± SD. Non-normally distributed data were presented using median (25th, 75th percentile). Abbreviations: ALB, albumin; *α*1-MG, *α*1-microglobulin; *β*2-MG, *β*2-microglobulin; CysC, cystatin C; Cr, creatinine; eGFR, estimated glomerular filter rate; KIM-1, kidney injury molecular 1; NAG, N-acetyl-*β*-(D)-glucosaminidase.

**Table 2:** Comparison of biochemical parameters and biomarkers of kidney injury in the study subjects.

	Control group (n = 92)	Group A (n = 40)	Group B (n = 33)	Group C (n = 19)
Blood lead (*μ*g/L)	33 [29, 38.75]	122.5 [95.25, 158.50]^*^	135 [93, 164.5]^*^	111 [76, 142]^*^
Urinary KIM-1/Cr (ng/g)	13.85 [9.20, 20.84]	27.25 [18.84, 40.24]^*^	36.16 [19.45, 54.79]^*^	28.34 [20.40, 50.70]^*^
Urinary NAG/Cr (U/g)	13.10 ± 5.98	20.51 ± 6.72^*^	18.42 ± 6.74^*^	19.99 ± 11.95^*^
Urinary α1-MG/Cr (mg/g)	4.35 [3.07, 7.09]	5.28 [3.82, 8.36]	6.87 [4.19, 8.88]^*^	7.85 [3.99, 9.26]^*^
Urinary β2-MG/Cr (mg/g)	0.13 [0.10, 0.21]	0.20 [0.15, 0.27]^*^	0.19 [0.12, 0.34]^*^	0.19 [0.13, 0.32]^*^

Note: Normally distributed data were presented as mean ± SD. Non-normally distributed data were presented using median (25th, 75th percentile). *p < 0.05 compared with control group.

**Table 3:** Correlation between blood lead levels and various indexes in the study subjects.

	Correlation coefficient (r)	*p* value
Age (year)	0.025	0.737
eGFR (ml/min × 1.73 m^2^)	−0.090	0.227
Serum CysC (mg/L)	0.722	0.001
Serum uric acid (*μ*mol/L)	0.003	0.965
Serum urea nitrogen (mmol/L)	0.099	0.179
Serum creatinine (mmol/L)	0.21	0.004
Urinary ALB/Cr	0.079	0.289
Urinary KIM-1/Cr (ng/g)	0.407	0.001
Urinary NAG/Cr (U/g)	0.349	0.001
Urinary *α*1-MG/Cr (mg/g)	0.194	0.008
Urinary *β*2-MG/Cr (mg/g)	0.215	0.003

**Table 4:** Area under the ROC curve for the analysis of urinary biomarkers in the lead-exposed subjects.

Tested Variable (s)	Area	Std. Error	Asymptotic Sig.	Asymptotic 95% Confidence Interval	
				Lower Bound	Upper Bound
Urinary KIM-1/Cr	0.819	0.031	0.001	0.758	0.880
Urinary NAG/Cr	0.788	0.034	0.001	0.721	0.856
Urinary *α*1-MG/Cr	0.634	0.041	0.002	0.554	0.715
Urinary *β*2-MG/Cr	0.669	0.040	0.001	0.591	0.748
Combined Index	0.890	0.024	0.001	0.842	0.938

Note: Combined Index includes urinary KIM-1/Cr, urinary NAG/Cr, urinary α1-MG/Cr and urinary β2-MG/Cr.

**Table 5:** Area under the ROC curve for the analysis of urinary KIM-1 or Combined Index in the lead-exposed subjects.

	Predicted probability	
Most Extreme Differences	Absolute	0.315
	Positive	0.315
	Negative	−0.109
Kolmogorov-Smirnov Z		2.138
Asymp. Sig. (2-tailed)		0.001
